# *N*-[(1*Z*)-Cyclo­dec-5-yn-1-yl­idene]hydroxyl­amine

**DOI:** 10.1107/S2414314625004146

**Published:** 2025-05-13

**Authors:** Heiner Detert, Dieter Schollmeyer

**Affiliations:** a University of Mainz, Department of Chemistry, Duesbergweg 10-14, 55099 Mainz, Germany; Goethe-Universität Frankfurt, Germany

**Keywords:** crystal structure, heterocycle, selenium, medium-sized ring

## Abstract

The crystal structure of cyclo­decynone oxime, C_10_H_15_NO, is reported. Two twist-boat-shaped cyclo­alkynes are centrosymmetrically connected *via* oxime hydrogen bridges. Deformation of the alkyne unit results from ring strain.

## Graphical abstract

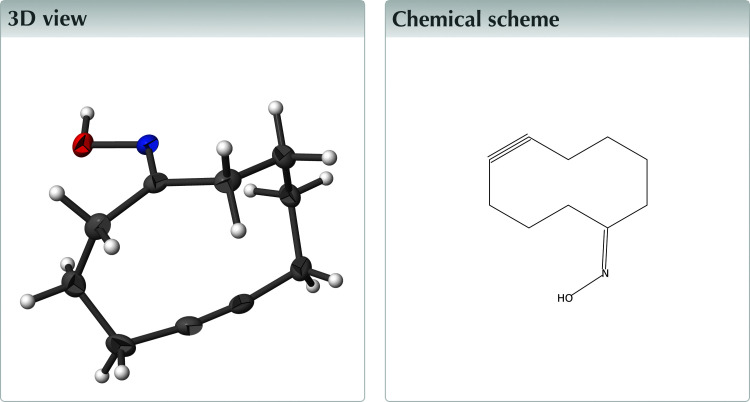

## Structure description

**Figure 1:**
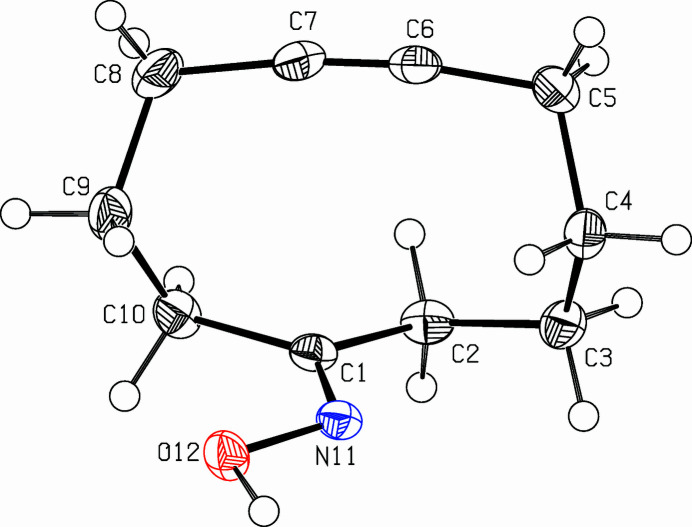
Perspective view (Spek, 2009 ▸) of the title compound. Displacement ellipsoids are drawn at the 50% probability level.

**Figure 2:**
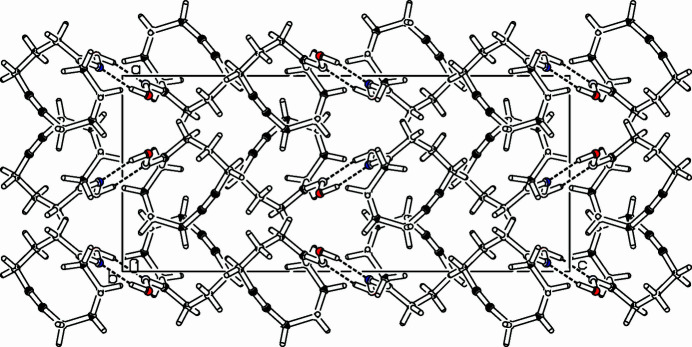
Part of the packing diagram. View along *b*-axis direction (Spek, 2009 ▸).

The title compound, C_10_H_15_NO (Fig. 1 ▸), was prepared as part of a project focusing on medium-sized rings and transannular reactions. Whereas the bond angle of 119.76 (9)° at the carbonyl group (C2—C1—C10) is perfect for a *sp*^2^-hybridized carbon, the C—C—C bond angles on the methyl­ene tether are significantly larger than for an ideal *sp*^3^ hybridization. Except for the propargylic carbon atoms C4—C5—C6: 111.67 (9)°, C7—C8—C9: 112.64 (9)°, the C—C—C bond angles vary between 114.75 (10)° and 116.98 (9)°. Ring strain also distorts the alkyne unit, bond angles on the acetyl­enic carbons are reduced to 172.02 (11)° for C5—C6—C7 and to 172.38 (11)° for C6—C7—C8. The cyclo­decyne ring adopts a twist-boat conformation with C4 and C9 being fore and aft. The other atoms are mostly coplanar, only C1 lies 0.477 (1) Å above and C2 lies −0.5213 (11) Å below this plane. The oxime unit (C1, N11, O12, H122) is planar with an r.m.s. deviation of 0.022 Å. Centrosymmetric dimers are formed *via* hydrogen bridging. The oxime units form a planar six-membered ring *via* two hydrogen bridges O12—H122—N11^i^, H122⋯N11^i^: 1.908 (19) Å (symmetry code as in Table 1 ▸) This gives the dimer the shape of two steps in a staircase (Fig. 2 ▸), the angle between the cyclo­decyne planes and the di-oxime plane being 75.1 (5)°.

## Synthesis and crystallization

Synthetic and spectroscopic details:

The title compound was prepared by G. Krämer (Krämer, 1996 ▸; Krämer *et al.*, 2009 ▸). Oxidation of deca­line to the hydro­peroxide, rearrangement to hy­droxy­ketone/hemiacetal and conversion *via* semicarbazone to 1,2,3-selena­diazole (Detert *et al.*, 1992 ▸), oxidation and pyrolysis yielded cyclo­decynone (Gleiter *et al.*, 1988 ▸). The oxime was formed according to Hanack (Hanack *et al.*, 1972 ▸).

The annotation of the NMR signals follows IUPAC nomenclature. ^1^H-NMR (200 MHz, CDCl_3_): 9.1 (*bs*, 1 H, OH), 2.75 (*t*, 2 H, *J* = 6.1 Hz), 2.37 (*t*, 2 H, *J* = 6 Hz), 2.20-1.95 (*m*, 6 H, 3,4,7-H), 1.80 (*m*, 4 H, 8,9-H); ^13^C-NMR (100 MHz, CDCl_3_): 160.3 (C=N), 84.9, 83.4 (C-5, C-6), 33.5, 30.6, 26.1, 24.3, 23.9, 19.7, 18.3.

## Refinement

Crystal data, data collection and structure refinement details are summarized in Table 2 ▸.

## Supplementary Material

Crystal structure: contains datablock(s) I, global. DOI: 10.1107/S2414314625004146/bt4170sup1.cif

Structure factors: contains datablock(s) I. DOI: 10.1107/S2414314625004146/bt4170Isup2.hkl

Supporting information file. DOI: 10.1107/S2414314625004146/bt4170Isup3.cml

CCDC reference: 2449294

Additional supporting information:  crystallographic information; 3D view; checkCIF report

## Figures and Tables

**Table 1:** Hydrogen-bond geometry (Å, °)

*D*—H⋯*A*	*D*—H	H⋯*A*	*D*⋯*A*	*D*—H⋯*A*
O12—H122⋯N11^i^	0.971 (19)	1.908 (19)	2.8030 (12)	151.9 (16)

Symmetry code: (i) 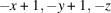.

**Table 2:** Experimental details

Crystal data	
Chemical formula	C_10_H_15_NO
*M*_r_	165.23
Crystal system, space group	Orthorhombic, *Pbca*
Temperature (K)	120
*a*, *b*, *c* (Å)	9.5469 (4), 8.9830 (3), 21.8191 (7)
*V* (Å^3^)	1871.20 (12)
*Z*	8
Radiation type	Mo *K*α
μ (mm^−1^)	0.08
Crystal size (mm)	0.80 × 0.48 × 0.14

Data collection	
Diffractometer	Stoe IPDS 2T
No. of measured, independent and observed [*I* > 2σ(*I*)] reflections	6155, 2574, 2225
*R*_int_	0.023
(sin θ/λ)_max_ (Å^−1^)	0.691

Refinement	
*R*[*F*^2^ > 2σ(*F*^2^)], *wR*(*F*^2^), *S*	0.042, 0.111, 1.04
No. of reflections	2574
No. of parameters	162
H-atom treatment	All H-atom parameters refined
Δρ_max_, Δρ_min_ (e Å^−3^)	0.37, −0.17

Computer programs: *X-AREAWinXpose*, *Recipe* and *Integrate* (Stoe & Cie, 2020 ▸), *SHELXT2014* (Sheldrick, 2015*a* ▸), *SHELXL2019/2* (Sheldrick, 2015*b* ▸) and *PLATON* (Spek, 2009 ▸).

## full crystallographic data

### N-[(1Z)-Cyclodec-5-yn-1-ylidene]hydroxylamine Crystal data

**Table:**

C_10_H_15_NO	*D*_x_ = 1.173 Mg m^−^^3^
*M**_r_* = 165.23	Mo *K*α radiation, λ = 0.71073 Å
Orthorhombic, *P**b**c**a*	Cell parameters from 9411 reflections
*a* = 9.5469 (4) Å	θ = 2.8–29.9°
*b* = 8.9830 (3) Å	µ = 0.08 mm^−^^1^
*c* = 21.8191 (7) Å	*T* = 120 K
*V* = 1871.20 (12) Å^3^	Plate, colorless
*Z* = 8	0.80 × 0.48 × 0.14 mm
*F*(000) = 720	

### N-[(1Z)-Cyclodec-5-yn-1-ylidene]hydroxylamine Data collection

**Table:**

Stoe IPDS 2T diffractometer	2225 reflections with *I* > 2σ(*I*)
Radiation source: sealed X-ray tube, 12x0.4mm long-fine focus	*R*_int_ = 0.023
Detector resolution: 6.67 pixels mm^-1^	θ_max_ = 29.4°, θ_min_ = 2.8°
rotation method, ω scans	*h* = −11→13
6155 measured reflections	*k* = −12→10
2574 independent reflections	*l* = −30→25

### N-[(1Z)-Cyclodec-5-yn-1-ylidene]hydroxylamine Refinement

**Table:**

Refinement on *F*^2^	Primary atom site location: dual
Least-squares matrix: full	Hydrogen site location: difference Fourier map
*R*[*F*^2^ > 2σ(*F*^2^)] = 0.042	All H-atom parameters refined
*wR*(*F*^2^) = 0.111	*w* = 1/[σ^2^(*F*_o_^2^) + (0.0486*P*)^2^ + 0.7721*P*] where *P* = (*F*_o_^2^ + 2*F*_c_^2^)/3
*S* = 1.04	(Δ/σ)_max_ < 0.001
2574 reflections	Δρ_max_ = 0.37 e Å^−^^3^
162 parameters	Δρ_min_ = −0.17 e Å^−^^3^
0 restraints	

### N-[(1Z)-Cyclodec-5-yn-1-ylidene]hydroxylamine Special details

**Table:**

Geometry. All esds (except the esd in the dihedral angle between two l.s. planes) are estimated using the full covariance matrix. The cell esds are taken into account individually in the estimation of esds in distances, angles and torsion angles; correlations between esds in cell parameters are only used when they are defined by crystal symmetry. An approximate (isotropic) treatment of cell esds is used for estimating esds involving l.s. planes.
Refinement. All hydrogen atoms were located in a difference map. The H atom bonded to O was freely refined. The coordinates of the H atoms attached to carbon atoms were freely refined. Their displacement parameters were also refined constraining the *U* values of each pair of H atoms bonded to the same C atom to be equal.

### N-[(1Z)-Cyclodec-5-yn-1-ylidene]hydroxylamine Fractional atomic coordinates and isotropic or equivalent isotropic displacement parameters (Å^2^)

**Table:**

	*x*	*y*	*z*	*U*_iso_*/*U*_eq_	
C1	0.58239 (10)	0.31386 (12)	0.09451 (4)	0.0202 (2)	
C2	0.53594 (12)	0.15439 (12)	0.08775 (5)	0.0239 (2)	
H2A	0.5224 (16)	0.1130 (17)	0.1307 (7)	0.035 (3)*	
H2B	0.6203 (17)	0.0993 (17)	0.0708 (7)	0.035 (3)*	
C3	0.40563 (12)	0.12659 (12)	0.04840 (5)	0.0265 (2)	
H3A	0.4264 (15)	0.1549 (17)	0.0047 (7)	0.034 (3)*	
H3B	0.3876 (16)	0.0187 (17)	0.0483 (7)	0.034 (3)*	
C4	0.27114 (12)	0.20571 (13)	0.06758 (5)	0.0269 (2)	
H4A	0.1977 (16)	0.1686 (17)	0.0414 (7)	0.034 (3)*	
H4B	0.2806 (16)	0.3164 (18)	0.0633 (7)	0.034 (3)*	
C5	0.22458 (13)	0.17707 (15)	0.13387 (6)	0.0312 (3)	
H5A	0.1284 (18)	0.2109 (19)	0.1418 (8)	0.044 (3)*	
H5B	0.2294 (18)	0.072 (2)	0.1442 (7)	0.044 (3)*	
C6	0.31476 (12)	0.25459 (12)	0.17804 (5)	0.0253 (2)	
C7	0.39879 (12)	0.32077 (13)	0.20810 (5)	0.0253 (2)	
C8	0.51520 (14)	0.39742 (16)	0.23847 (5)	0.0337 (3)	
H8A	0.5657 (18)	0.3253 (19)	0.2646 (8)	0.046 (3)*	
H8B	0.4801 (19)	0.4749 (19)	0.2667 (8)	0.046 (3)*	
C9	0.61843 (12)	0.46629 (14)	0.19295 (5)	0.0280 (2)	
H9A	0.6953 (15)	0.5097 (16)	0.2174 (7)	0.031 (3)*	
H9B	0.5754 (15)	0.5494 (16)	0.1713 (7)	0.031 (3)*	
C10	0.67983 (11)	0.35670 (13)	0.14614 (5)	0.0249 (2)	
H10A	0.7644 (16)	0.4019 (17)	0.1266 (6)	0.033 (3)*	
H10B	0.7124 (15)	0.2666 (17)	0.1673 (7)	0.033 (3)*	
N11	0.54369 (9)	0.40276 (10)	0.05199 (4)	0.02058 (19)	
O12	0.60094 (8)	0.54785 (9)	0.05794 (4)	0.02628 (19)	
H122	0.5675 (19)	0.595 (2)	0.0207 (9)	0.054 (5)*	

### N-[(1Z)-Cyclodec-5-yn-1-ylidene]hydroxylamine Atomic displacement parameters (Å^2^)

**Table:**

	*U*^11^	*U*^22^	*U*^33^	*U*^12^	*U*^13^	*U*^23^
C1	0.0171 (4)	0.0245 (5)	0.0190 (4)	0.0022 (4)	0.0032 (3)	0.0029 (4)
C2	0.0263 (5)	0.0209 (5)	0.0247 (5)	0.0050 (4)	0.0043 (4)	0.0016 (4)
C3	0.0315 (6)	0.0224 (5)	0.0256 (5)	−0.0018 (4)	0.0027 (4)	−0.0059 (4)
C4	0.0238 (5)	0.0308 (6)	0.0262 (5)	−0.0040 (4)	−0.0017 (4)	−0.0060 (4)
C5	0.0279 (6)	0.0333 (6)	0.0324 (6)	−0.0104 (5)	0.0077 (5)	−0.0064 (5)
C6	0.0275 (5)	0.0268 (5)	0.0214 (5)	−0.0009 (4)	0.0082 (4)	0.0014 (4)
C7	0.0276 (5)	0.0311 (5)	0.0170 (4)	0.0023 (4)	0.0035 (4)	0.0018 (4)
C8	0.0328 (6)	0.0496 (7)	0.0186 (5)	−0.0020 (6)	−0.0046 (4)	−0.0022 (5)
C9	0.0273 (5)	0.0317 (6)	0.0251 (5)	−0.0036 (5)	−0.0084 (4)	0.0012 (4)
C10	0.0192 (5)	0.0302 (5)	0.0254 (5)	−0.0002 (4)	−0.0048 (4)	0.0077 (4)
N11	0.0209 (4)	0.0218 (4)	0.0190 (4)	−0.0011 (3)	0.0023 (3)	0.0027 (3)
O12	0.0287 (4)	0.0248 (4)	0.0253 (4)	−0.0075 (3)	−0.0046 (3)	0.0079 (3)

### N-[(1Z)-Cyclodec-5-yn-1-ylidene]hydroxylamine Geometric parameters (Å, º)

**Table:**

C1—N11	1.2785 (13)	C5—H5B	0.969 (18)
C1—C2	1.5069 (15)	C6—C7	1.1946 (16)
C1—C10	1.5108 (14)	C7—C8	1.4656 (16)
C2—C3	1.5319 (16)	C8—C9	1.5298 (17)
C2—H2A	1.017 (15)	C8—H8A	0.989 (17)
C2—H2B	1.015 (16)	C8—H8B	0.988 (17)
C3—C4	1.5260 (16)	C9—C10	1.5349 (17)
C3—H3A	1.007 (15)	C9—H9A	0.987 (15)
C3—H3B	0.984 (15)	C9—H9B	0.974 (15)
C4—C5	1.5349 (16)	C10—H10A	0.999 (15)
C4—H4A	0.964 (15)	C10—H10B	0.983 (15)
C4—H4B	1.003 (16)	N11—O12	1.4193 (11)
C5—C6	1.4680 (16)	O12—H122	0.971 (19)
C5—H5A	0.983 (17)		
			
N11—C1—C2	115.96 (9)	C4—C5—H5B	111.6 (10)
N11—C1—C10	124.04 (10)	H5A—C5—H5B	107.7 (14)
C2—C1—C10	119.76 (9)	C7—C6—C5	172.02 (11)
C1—C2—C3	116.67 (9)	C6—C7—C8	172.38 (11)
C1—C2—H2A	107.1 (9)	C7—C8—C9	112.64 (9)
C3—C2—H2A	110.7 (9)	C7—C8—H8A	108.8 (10)
C1—C2—H2B	105.4 (9)	C9—C8—H8A	109.0 (10)
C3—C2—H2B	111.2 (9)	C7—C8—H8B	110.8 (10)
H2A—C2—H2B	104.9 (12)	C9—C8—H8B	109.8 (10)
C4—C3—C2	116.98 (9)	H8A—C8—H8B	105.5 (14)
C4—C3—H3A	107.9 (9)	C8—C9—C10	114.75 (10)
C2—C3—H3A	109.3 (9)	C8—C9—H9A	106.7 (9)
C4—C3—H3B	108.2 (9)	C10—C9—H9A	109.2 (9)
C2—C3—H3B	107.7 (9)	C8—C9—H9B	110.6 (9)
H3A—C3—H3B	106.3 (12)	C10—C9—H9B	109.3 (9)
C3—C4—C5	115.09 (10)	H9A—C9—H9B	105.8 (12)
C3—C4—H4A	106.8 (9)	C1—C10—C9	115.11 (9)
C5—C4—H4A	106.8 (9)	C1—C10—H10A	106.5 (8)
C3—C4—H4B	111.1 (9)	C9—C10—H10A	109.4 (9)
C5—C4—H4B	106.3 (8)	C1—C10—H10B	109.6 (9)
H4A—C4—H4B	110.6 (12)	C9—C10—H10B	109.6 (9)
C6—C5—C4	111.67 (9)	H10A—C10—H10B	106.3 (12)
C6—C5—H5A	106.6 (10)	C1—N11—O12	113.33 (8)
C4—C5—H5A	112.7 (10)	N11—O12—H122	101.5 (11)
C6—C5—H5B	106.3 (10)		
			
N11—C1—C2—C3	−23.67 (13)	N11—C1—C10—C9	69.25 (13)
C10—C1—C2—C3	161.71 (9)	C2—C1—C10—C9	−116.59 (11)
C1—C2—C3—C4	−57.70 (13)	C8—C9—C10—C1	76.41 (12)
C2—C3—C4—C5	−55.44 (14)	C2—C1—N11—O12	−174.64 (8)
C3—C4—C5—C6	72.79 (13)	C10—C1—N11—O12	−0.28 (14)
C7—C8—C9—C10	−55.59 (14)		

### N-[(1Z)-Cyclodec-5-yn-1-ylidene]hydroxylamine Hydrogen-bond geometry (Å, º)

**Table:**

*D*—H···*A*	*D*—H	H···*A*	*D*···*A*	*D*—H···*A*
O12—H122···N11^i^	0.971 (19)	1.908 (19)	2.8030 (12)	151.9 (16)

Symmetry code: (i) −*x*+1, −*y*+1, −*z*.
